# Codoping and Interstitial Deactivation in the Control of Amphoteric Li Dopant in ZnO for the Realization of p-Type TCOs

**DOI:** 10.3390/ma10040332

**Published:** 2017-03-23

**Authors:** Alessandra Catellani, Arrigo Calzolari

**Affiliations:** CNR-NANO Istituto Nanoscienze, Centro S3, via Campi 213A, I-41125 Modena, Italy; alessandra.catellani@nano.cnr.it

**Keywords:** TCO, ZnO, p-doping, codoping

## Abstract

We report on first principle investigations about the electrical character of Li-X codoped ZnO transparent conductive oxides (TCOs). We studied a set of possible X codopants including either unintentional dopants typically present in the system (e.g., H, O) or monovalent acceptor groups, based on nitrogen and halogens (F, Cl, I). The interplay between dopants and structural point defects in the host (such as vacancies) is also taken explicitly into account, demonstrating the crucial effect that zinc and oxygen vacancies have on the final properties of TCOs. Our results show that Li-ZnO has a p-type character, when Li is included as Zn substitutional dopant, but it turns into an n-type when Li is in interstitial sites. The inclusion of X-codopants is considered to deactivate the n-type character of interstitial Li atoms: the total Li-X compensation effect and the corresponding electrical character of the doped compounds selectively depend on the presence of vacancies in the host. We prove that LiF-doped ZnO is the only codoped system that exhibits a p-type character in the presence of Zn vacancies.

## 1. Introduction

Transparent conducting oxides (TCOs) are a special class of materials that combine electrical conductivity with transparency for visible light [1]. They play a key role in optoelectronic applications [2] such as photovoltaics [3], touch-screen sensors, low emissivity windows, liquid crystal display (LCD) devices, plasma and organic light emitting diode (OLED) displays, and smart windows. Prototype TCO semiconductors are impurity-doped ZnO, In${}_{2}$O${}_{3}$, SnO${}_{2}$ and CdO, and multi-component oxides consisting of combinations of them. Sn doped In${}_{2}$O${}_{3}$ (ITO), F doped SnO${}_{2}$ and Al doped ZnO (AZO) TCO thin films are the materials of choice for most of the present applications.

Most of the optical and electronic properties of TCOs derive from the characteristic band structure of the host. First, the requirement of transparency corresponds to wide bandgap E${}_{g}>$ 3.0 eV. The second desired characteristic is a low electron effective mass [4], in order to assure a high electron mobility and thus good conduction [5]. In TCOs, the low effective mass results from a unique almost parabolic conduction band, usually with a deep minimum at Γ. This corresponds to a very low density of states in the conduction band bottom, so that the average gap over the entire Brillouin zone is remarkably higher than the optical gap. The mid point of this average gap is known as *charge neutrality level* (CNL) and defines p- and n-dopability of a semiconductor. Indeed, n-type doping is difficult if the conduction band edge lies too far above CNL, and p-type doping becomes difficult if the valence band edge lies too deep below CNL. In semiconductors, usually CNL lies in the middle of the optical gap. In TCOs, CLS is degenerate or deep in the conduction band, and actually these compounds are intrinsically n-type. Intrinsic n-donor defects can be associated with oxygen vacancies or cation interstitials, as well as unintentional incorporation of residual species (H, O, etc.) present in the sample. On the other hand, since CNL is so far above the valence band edge, p-type conductivity is almost absent in TCOs. Nonetheless p-type materials are a desired requirement for many basic electronics. In particular, the possibility of obtaining both p- and n-type TCOs by selectively changing the doping of the same host would help the integration in the final device, reducing mismatches and interfaces. This explains the huge effort provided in the research of p-type TCOs [6,7,8]. Although a few p-type TCOs have already made their way into optoelectronic devices (e.g., copper delafossites), current p-type TCOs are still unable to match the performance of n-type TCOs in terms of charge carrier mobility and/or optical transparency.

Particularly interesting is the case of lithium doping in ZnO. Lithium has been considered both as an external dopant included in ZnO through several post-growth techniques (such as sol gel technology, electro deposition, vapour phase deposition, magnetron DC sputtering, chemical vapour deposition, electron beam evaporation, pulsed laser deposition, etc.) [9,10,11] and as unintentional residual doping in ZnO samples grown via hydrothermal or chemical synthesis techniques [12,13,14]. One ultimate challenge is related to the amphoteric nature of Li, since Li on an interstitial site acts as a donor, leading to self-compensation. Thus, codoping with electron acceptor groups has been proposed as a strategy to deactivate the donor capability of interstitial Li atoms and control the electrical properties of the host [15,16,17]. The large variability of the degrees of freedom (such as doping techniques, quality of the sample, chemical environments, temperature and pressure, etc.) makes it difficult to obtain a coherent view from the plethora of experimental data, hindering a deep comprehension of the role of the single actors (e.g., dopants, defects, host) on the final electrical character of the system.

Here, we present an extensive study, based on ab initio simulations, of the Li-X codoping of ZnO. We considered a large range of X codopants that include unintentional dopants and monovalent acceptor groups, based on nitrogen and halogens (F, Cl, I). Since the adsorption site of Li (e.g., substitutional vs. interstitial) is crucial in the definition of the final electrical properties of the sample, we considered both configurations, and we evaluated the interplay between dopants and structural point defects (zinc, oxygen, and Zn-O dimer vacancies) that may otherwise be present in the ZnO host. Our results show that the realization of p-type TCO is a complicated task that requires a fine tuning of the localization and compensation effects. Among the considered cases, only two Li configurations exhibit a p-type character, the remaining leading to n-type TCOs. Halogens seem the best candidates to deactivate the donor properties of interstitial Li, restoring a charge neutrality in the system.

## 2. Results

**Figure 1:**
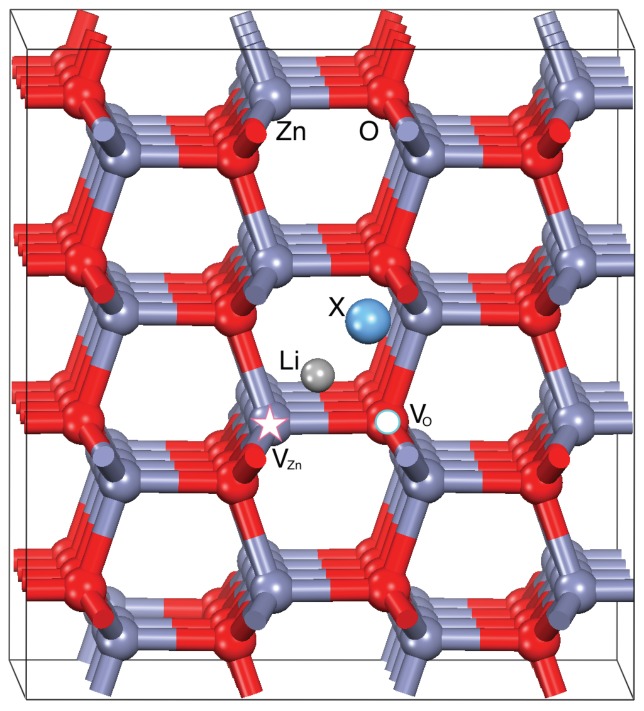
3D atomic structure of initial Li-X doped ZnO (LXZO) geometries. Grey (red) balls indicate Zn (O) atoms. Labels Li and X and correspondingly light grey and blue spheres identify the different dopant chemical species. V${}_{O}$ and V${}_{Zn}$ hosts are obtained by removing the atoms marked by the circle and the star, respectively; V${}_{dim}$ host results from the removal of both atoms.

We considered the experimental conditions where the dopants are included in existing (i.e., previously prepared) ZnO samples through post-growth techniques, such as evaporation and magneto sputtering. This being the case, the inclusion of dopants does not compete with the formation of structural defects in the ZnO host during the growth processes. Thus, we can realistically assume that the undoped ZnO samples intrinsically include structural defects, such as vacancies and dislocations, whose amount strictly depends on the growth techniques. In order to decouple the complex interplay between the existing defects and the dopants, we considered four possible initial hosts each including zero (ideal ZnO crystal) or one neutral point defect, namely: an oxygen (V${}_{O}$), a zinc (V${}_{Zn}$), and a Zn-O dimer (V${}_{dim}$) vacancy, as shown in Figure 1. All defective ZnO structures were fully relaxed to the minimum energy configurations. Electronic structures of the resulting systems well agree with previous calculations [18,19,20]: undefective ZnO has a semiconducting behavior with the Fermi level lying in the middle of the bandgap (E${}_{g}^{DFT+U}$ = 3.2 eV). V${}_{O}$ is a deep defect, which introduces fully occupied states in the bandgap. In contrast, Zn vacancy is a dominant acceptor in ZnO that introduces shallow defect states close to the valence band maximum (VBM) [21]. The electronic effects due to V${}_{O}$ and V${}_{Zn}$ compensate in the case of the removal of a Zn-O dimer, which does not exhibit defect states in the energy gap, similar to ideal ZnO. From here on, we refer to V${}_{O}$, V${}_{Zn}$, and V${}_{dim}$ configurations as the ZnO bulk host including the corresponding point defect. Accordingly, here ZnO, V${}_{O}$, V${}_{Zn}$, V${}_{dim}$ configurations constitute the four reference hosts for Li-X doping.

Lithium doping is simulated by adding one Li atom per cell. This corresponds to a formal Li concentration equal to ∼0.8 at.%. Then, we considered the effect of Li-X codoping, through the co-inclusion of chemical groups (X). The choice of Xs has been made considering either unintentional dopants typically present in the system (e.g., H, O) that can combine with Li, or monovalent acceptor units, based on nitrogen and halogen, that can compensate lithium or interact with the oxygen vacancies of the host. Finally, among possible X dopants, we selected groups that might form stable LiX molecules with atomic lithium. We distinguished four Li-X groups: (i) dopants including only Li and/or H: atomic Li, Li-Li (i.e., Li${}_{2}$) and Li-H; (ii) dopants including oxygen: Li-OH, Li-LiO (i.e., Li${}_{2}$O); (iii) dopants including nitrogen Li-N, Li-NH${}_{2}$, Li-NO${}_{2}$, and Li-CN; and (iv) dopants including halogens Li-F, Li-Cl, and Li-I. We combined the 12 Li-X pairs with the four ZnO hosts to obtain different kinds (i.e., 48) of Li-X doped ZnO (hereafter, LXZO) compounds.

The initial geometry for each LXZO system has been obtained including an Li atom and a X codopant in interstitial positions with respect to the crystalline host, which may include a relaxed point defect in the structure (Figure 1). Every LXZO system has been first heated to room temperature for 1.5 ps (via ab initio molecular dynamic simulations), and then relaxed to the ground state at T = 0 K. Finally, the DFT+U (see Section 4) electronic structure has been collected for the optimized samples. Despite the details specific of single configurations, some common features may be highlighted: (1) bonded LiX molecules never form and Li and X separately interact with the host; (2) Li systematically occupies the zinc vacancy, whenever present (V${}_{Zn}$ and V${}_{dim}$ substrates), assuming a final Zn substitutional position (Li${}_{Zn}$) in the crystal, while it never occupies the oxygen sites, in agreement with the experimental results [22]; (3) when V${}_{Zn}$ sites are not available (V${}_{O}$, and ZnO substrates), Li remains in an interstitial position (Li${}_{I}$) at the average distance of ∼2.3 Å (∼1.9 Å) from nearest-neighbor oxygen (zinc) atoms, respectively; (4) codopants including oxygen, nitrogen or halogens easily occupy the oxygen vacancies, whenever present; and (5) in the limit of the time and temperature range considered in the simulations, neither Li nor X is able to remove and replace an atom from the pristine crystal. Notably, previous molecular dynamics simulations on Al:ZnO systems [23] exhibited the easy Zn removal from original crystalline sites upon interstitials Al defects. This remarkable difference between Li and Al is probably due to stronger affinity of Al for oxygen to form Al${}_{2}$O${}_{3}$ stable phase.

**Figure 2:**
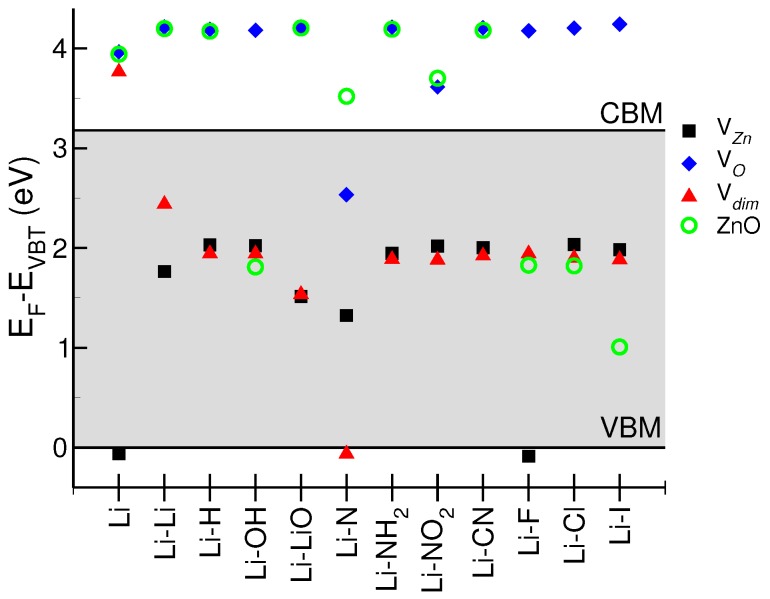
Final energy position of Fermi level (E${}_{F}$) of LXZO systems as a function of the four considered host substrates (ZnO, V${}_{O}$, V${}_{Zn}$, V${}_{dim}$). Zero energy reference is set to valence band maximum of undoped and undefective ZnO bulk. Gray shaded area identifies the ZnO bandgap. In the case of ideal ZnO crystal E${}_{F}$ lies in the middle of the bandgap.

Figure 2 summarizes the electrical behavior of the resulting LXZO systems. For each Li-X pair (*x*-axis), and, for each substrate, the colored symbols represent the energy position of the Fermi level (E${}_{F}$) with respect to the bandgap (E${}_{g}$, gray shaded area) of the ideal undoped ZnO, whose valence band maximum (VBM) is assumed as zero energy reference. Systems with E${}_{F}$ above the conduction band minimum (CBM) of ZnO act as degenerate n-type conductors. On the contrary, systems whose E${}_{F}$ is below VBM represent degenerate p-type conductors. Finally, systems with E${}_{F}$ lying in the bandgap are neutral semiconductors.

Only two configurations, namely, Li(V${}_{Zn}$) and Li-F(V${}_{Zn}$), exhibit a net p-type character; in both cases, Li is Zn substitutional. The p-type character Li${}_{Zn}$:ZnO is generally neutralized by the presence of X-codopants. Thus, the other systems including Li${}_{Zn}$ (V${}_{Zn}$ and V${}_{dim}$ substrates) generally have a neutral semiconducting behavior.

Interstitial lithium (V${}_{O}$ and ZnO substrates) is responsible for an n-type character of most LXZO compounds. In particular, except for Li-N(V${}_{O}$), the presence of oxygen vacancies in the host is always associated to an n-type character of the resulting LXZO sample. The accidental presence of codopants (such as OH), which may saturate the original oxygen vacancy, does not change the picture. Similar results are observed also for highly crystalline hosts with negligible intrinsic defects (e.g., ZnO); except for halogen ions that exactly compensate the Li${}_{I}$ charge, in all of the other cases, the absence of defect states in the bandgap of the host facilitates the direct charge injection from Li to CBM. We can conclude that the presence of structural defects play a crucial role in the definition of the electrical character of Li-ZnO system, while X-codopants generally fail in the deactivation of Li${}_{I}$ donors. Halogens are the unique elements able to restore the charge neutrality in the presence of substitutional Li atoms.

**Figure 3:**
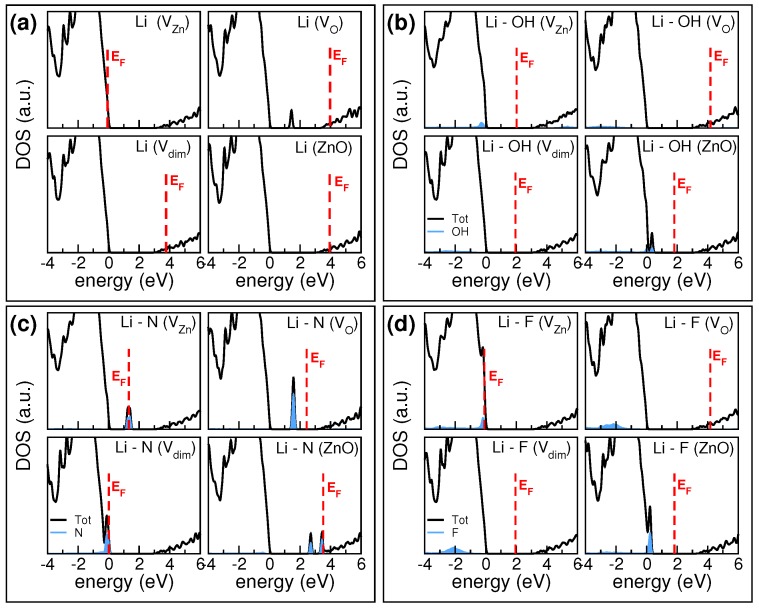
Density of states (DOS) plots of LXZO compounds for selected Li-X dopants: (**a**) Li; (**b**) Li-OH; (**c**) Li-N; (**d**) Li-F. For each dopant, DOSs corresponding to the four possible hosts are displayed. Zero energy reference is set to the valence band maximum of ZnO. Red vertical lines identify the resulting Fermi level of the doped systems. Cyan shaded areas represent the X-codopant contributions due to total DOS.

In order to gain insight on these dopant-defect compensation effects, we explicitly analyzed the electronic structures of a few selected Li-X pairs, representative of each Li-X dopant group, defined above. Figure 3 summarizes the total and X-projected density of states (DOS), for each host. In all cases, Li simply donates its 2s electron to the system; this is never associated to the appearance of an Li-derived peak in the DOS, at least in the gap region. Thus, only X-projected contributions are explicitly shown in Figure 3.

When no other impurities are present, Li atom (first column set on the left of Figure 2) is an amphoteric dopant for ZnO, whose final electrical behavior selectively depends on presence/absence of specific point defects. In the case of V${}_{Zn}$, although Li spatially occupies the Zn site, its unpaired *2s* electron is not able to fully saturate the defect of the 2-electron associated with the Zn vacancy. This results in a partial depletion of charge on the valence band, while no other defect states arise in the gap. Thus, the system acts as a degenerate p-type conductor, in agreement with many experimental results at low Li concentration [11,12,22,24]. The coexistence of hydrogen (e.g., Li-H) or high Li concentration (e.g., Li-Li) can saturate the charge unbalance due to the Zn vacancy, neutralizing the effect of Li${}_{Zn}$ [25]. In the other three cases (V${}_{O}$, V${}_{dim}$, ZnO), for which the initial valence band is fully occupied, Li${}_{I}$ donates its 2s electron to the first available empty states, i.e., the conduction band of the host which thus turns to n-type. This ambivalent n/p doping effect of Li has been observed experimentally by many authors who demonstrated that Li-ZnO switches from a p-type to an n-type conductor [9,12,17,26], increasing the amount of Li dopants. A similar effect is due to hydrogen that always acts a monovalent electron donor, which contributes with Li to inject a free charge into the host conduction band.

The modifications of the electronic structure deriving from the inclusion of OH codopants are shown in Figure 3b. In the case of the V${}_{Zn}$ host, Li spatially occupies the Zn vacancy site and the OH fragment dissociates: the oxygen remains interstitial and coordinates to two neighboring Zn atoms, deforming the crystalline order; hydrogen bonds to an O-atom of the host. Li${}_{Zn}$ and H saturate the charge deficiency of V${}_{Zn}$. The extra oxygen, being coordinated to Zn, becomes electronically indistinguishable from the other oxygens of the host. This generates the presence of a fully occupied state deep in the valence band of ZnO, while no defect states appear in the bandgap. The system behaves as a neutral semiconductor. The case of V${}_{dim}$ substrate is similar, with the only difference that the extra oxygen spatially saturates the oxygen vacancy, reducing the atomic distortion in the crystal. The neutralization of substitutional Li upon OH compensation has been experimentally observed, e.g., in Ref. [25]. In the other two cases, lithium that is not involved in saturating zinc vacancies donates its valence electron to the host, which assumes an n-type character.

Among the Li-X pair considered, codopants including nitrogen exhibit the most complex electronic structures, since they lead to the presence of defect states in the pristine ZnO forbidden gap. The case of Li-N is shown in panel (c). Nitrogen has one electron less than oxygen and it has been proposed as a p-type dopant for ZnO, when it replaces oxygens [10,27]. Differently from oxygen-based codopants, which have an obvious affinity with the ZnO environment, nitrogen is less electronegative than O and hence less capable to capture Zn-charge to form Zn-N bonds. Thus, the presence of N atoms induces the formation of less-bonded states (i.e., lower binding energy) than the corresponding oxygen ones, which constitute the VBM of clean ZnO. This results in the appearance of N-derived states in the bandgap, in which position and occupation depends on the total charge balance between Li and host vacancies. In the case of ZnO, V${}_{O}$, and V${}_{Zn}$ substrates, N induces deep defect states that are not associated to a donor/acceptor character of the doping, but are rather traps for charge transport. Defect states in the bandgap also affect the optical properties of the system, since they are responsible for optically active interband transitions in the visible range. Thus, the inclusion of N-derived elements deteriorates both the electrical conductivity and the transparency of the sample, which does not have the characteristics of a TCO anymore. In the special case of Li-N in V${}_{dim}$ substrate, Li and N occupy the zinc and oxygen vacancy sites, respectively. However, both substituents are electron deficient with respect to the corresponding replaced species. This generates a partially occupied peak at +200 meV from the top of the valence band (Figure 2). The final effect is the shift of the Fermi level close to the VBM. This, however, is not sufficient to make the system a degenerate p-type conductor, as for the pure Li${}_{Zn}$ doping. Li-N complex is more properly a deep acceptor as suggested by photoluminescence measurements [28].

Due to the strong electronegativity and their capability in forming ionic salt with alkaline metals, halogens are a natural choice to deactivate the donor activity (n-doping) of interstitial lithium atoms [15,17,29,30]. Figure 2 confirms this simple chemical intuition, at least for ZnO and V${}_{dim}$ substrates. When halogen and lithium are both substitutional or both interstitial, they simply compensate each other, without further modifying the electronic properties of the host that maintains its original semiconducting character. This is also evident from DOS plots of Figure 3d calculated in the specific case of Li-F codoping. Fluorine deserves a special insight: several experimental [31,32,33,34] and theoretical [35] works demonstrated an amphoteric character of fluorine in ZnO. Fluorine is a strong donor when absorbed in oxygen substitutional sites, while it behaves like a weak acceptor when included in the interstitial position. Even though isoelectronic to fluorine, other halogens exhibit only donor properties and cannot be used for p-type doping.

These features sum up in the case of co-doping with lithium. When Li is interstitial and F is substitutional as in the $V_{O}$ configuration (panel d), the system results a degenerate n-conductor, since both Li and F acts as donors. Similar results are observed for chlorine and iodine. When the opposite situation occurs, i.e., Li substitutional and halogen interstitial, we observe different electrical characteristics: Cl and I simply compensate the p-type character of the substitutional Li dopant giving a neutral system, while F preserves the p-type character of the sample. In the interstitial configuration, F approaches and binds to an oxygen atom of the host. Due to the stronger electronegativity of F with respect to O, this causes an O-to-F charge transfer and a partial charge reduction of the ZnO valence band. This, along with the partial saturation of zinc vacancy by Li${}_{Zn}$, induces a global p-type behavior to the system (panel d). All other halogen elements are less electronegative and are not able to oxydize the host and simply compensate the Li charge.

We now investigate the thermodynamic conditions for the dopability of the systems described above. In most cases, LXZO compounds behave either as (p/n) degenerate conductors or as compensated neutral semiconductors. In particular, since Li-X pairs do not introduce shallow defect states in the bandgap, we can restrict our analysis to the calculation of formation enthalpies of neutral impurities. The formation enthalpy ($\Delta H_{for}$) for Li-X in ZnO can be obtained as :

(1) $$\Delta H_{for}=E^{tot}-E_{\alpha }^{host}-\mu \left(Li\right)-\mu \left(X\right),$$

where $E^{tot}$ is the calculated total energy [36] for the relaxed LXZO compound, which includes the host and the Li-X impurity. $E_{\alpha }^{host}$ is the total energy of the initial substrate, and α specifies the actual V${}_{O}$, V${}_{Zn}$, V${}_{dim}$, and ZnO host. Following the hypothesis made at the beginning, that structural defects are already present in the sample, we do not consider the enthalpy contribution ($\Delta H_{for}^{def}$) due to the formation of vacancies. Thus, we assume as host reference energy $E_{\alpha }^{host}$ the total energy of the corresponding relaxed defective system. μ(Li) and μ(X) are the chemical potential for Li and X, respectively, with respect to their stable phase in the experimental conditions. Since oxygen and zinc are the constitutive elements of ZnO, and thus abundantly present in the system, the chemical potential of lithium and fluorine are referred to Li${}_{2}$O and ZnF${}_{2}$ bulk, which are the stable compounds of Li and F with oxygen and zinc, respectively. Li${}_{2}$O and ZnF${}_{2}$ are indeed the materials used experimentally to dope ZnO, through sputtering techniques [26,34]. Biatomic molecules H${}_{2}$, O${}_{2}$, N${}_{2}$, Cl${}_{2}$, and I${}_{2}$ are assumed as the references for the other chemical species. The CNH molecule is used to evaluate the chemical potential of CN unit.

**Figure 4:**
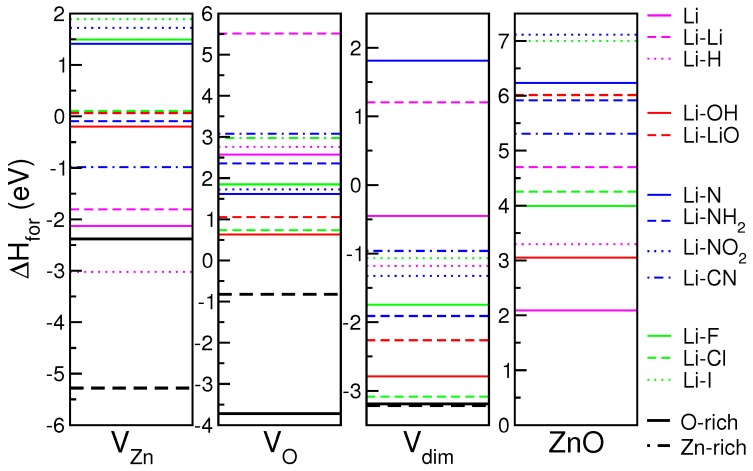
Formation enthalpies ($\Delta H_{for}$) of LXZO compounds. Each panel corresponds to an initial host, from left to right: V${}_{Zn}$, V${}_{O}$, V${}_{dim}$, ZnO. The color code (pink, red blue, green) distinguishes the four classes of Li-X dopants discussed in the text. In each panel, the zero energy is set to the total energy of the corresponding host system, taken as the local reference. Black lines mark $-\Delta H_{for}^{def}$, where $\Delta H_{for}^{def}$ is the formation enthalpy of the point defects (V${}_{O}$, V${}_{Zn}$, V${}_{dim}$) corresponding to O-rich (straight line) and Zn-rich (dashed line) growth conditions.

Formation enthalpies for the 12 Li-X codopants are reported in Figure 4, for each initial substrate. Notably, as the reference is different depending on the defect included in the host, the zero energy in the four panels of Figure 4 is not unique, but each is related to the corresponding $E_{\alpha }^{host}$ value. Thus, in each panel, negative (positive) values correspond to stable (unstable) systems with respect to the specific host.

In the case of V${}_{Zn}$ and V${}_{dim}$ substrates, most of the dopants can be easily included in the system, except for a few N- and halogen compounds. In this case, part of the energy gain derived from the saturation of the existent vacancy. On the contrary, none of the considered Li-X pair is energetically stable if V${}_{O}$ or undefective ZnO are considered as the reference. This agrees with the observation that, during the simulated annealing at room temperature, none of the impurities is able to replace one Zn or O atom from the bulk. These kinds of processes imply a higher activation barrier that can be furnished to the system changing the characteristics’ setups (e.g., temperature, pressure, flux intensity) during the experiment. Since the n-doping character of Li-ZnO is mostly due to interstitial adsorption sites, and since Li interstitial is energetically hardly stable (see V${}_{O}$ and ZnO panels), we conclude that Li n-doping is not a thermodynamically favored process, at least at low Li concentrations. On the other hand, if Li interstitials exist, oxygen based codopants seem to be the energetically favored elements to deactivate (i.e., compensate) the donor activity of lithium, restoring the neutral electrical character to LXZO compound.

## 3. Discussion

Although Li${}_{Zn}$ is responsible for the p-type electrical character of Li:ZnO, this does not mean that Li${}_{Zn}$ is an acceptor dopant for ZnO. In all considered cases, Li always donates its 2s electron to the host, either being in a substitutional or in an interstitial configuration. The observed p-conductivity derives, instead, from the partial saturation by lithium of the charge depletion associated to the Zn vacancy that is, indeed, the unique electron acceptor in the studied systems. More generally, the balance between the number of Zn vacancies and dopants would be responsible for the final charge character of realistic doped-defective systems.

When Li is adsorbed as interstitial, it imparts an n-type character to the host. We have already noted, in particular, that the presence of oxygen vacancies is always associated to an n-type character of the resulting LXZO sample. It is important to note that this behavior is not due to a presumed shallow donor character of V${}_{O}$, as sometimes proposed in previous works [37], but rather to the fact that Li and V${}_{O}$ do not directly interact. As discussed above, the presence of oxygen vacancies is responsible for deep and fully occupied defect states in the energy gap that are, however, not affected by the presence of Li, which donates its valence electron to the first available empty states.

Inclusion of codopants based on oxygen or halogen atoms are promising choices to compensate interstitial Li atoms restoring a semiconducting charge neutrality. The analysis of the formation enthalpies favors the inclusion of oxygen-derived codopants with respect to halogens. N-containing systems, having high formation enthalpies and inserting deep states in the bandgap, are instead not suitable for compensating Li-doped ZnO.

In the comparison of formation enthalpies (Figure 4), it is necessary to keep in mind that the choice of the stable phases assumed as reference in the evaluation of chemical potentials (Equation (1)) is not unique and strictly depends on the specific chemical environment perceived by the dopants during the experiments. For example, depending on the amount of O${}_{2}$ or water present in the sample, it may be more appropriate to refer the chemical potential of the OH codopant to H${}_{2}$O, rather than to the separated elements, as proposed above. In a similar way, if fluorine, instead of being injected from sputtering from the ZnF${}_{2}$ solid, is evaporated from the F${}_{2}$ molecule, the reference chemical potential should be modified accordingly. Conversely, as solid ZnCl${}_{2}$ and ZnI${}_{2}$ are not stable phases for heavy halogens, the biatomic molecule is the most natural reference. Different choices for the chemical potential μ(X) imply different formation enthalpies and thus a different order and reciprocal stability among the Li-X pairs. This, however, does not change the qualitative description given in the previous section.

In the analysis of stability, we did not consider the formation enthalpy of the single vacancies, as we supposed that defects were already present in the sample before the doping process. If doping is instead competitive with the formation of defects, as it happens, for instance, when dopants are provided during growth, the formation enthalpy of the single defects must be taken into account. Black lines in Figure 4 mark the values $-\Delta H_{for}^{def}$, where $\Delta H_{for}^{def}$ is the formation enthalpy of the point defects (V${}_{O}$, V${}_{Zn}$, V${}_{dim}$) corresponding to O-rich (straight line) and Zn-rich (dashed line) growth conditions. In this case, the ideal undefective ZnO crystal is the reference for all of the systems. If we assume this as the energy origin in Figure 4, lines below (above) black lines correspond to stable (unstable) configurations, respectively. Hence, except for Li-H, all the other systems would result in being energetically unstable, preventing the dopability of ZnO with Li. Li-doped compounds have, however, been experimentally realized: this confirms that structural defects play a direct role in thermodynamic stability of the dopants, while ideal undefective ZnO cannot be assumed as a realistic reference in the formation of LXZO compounds.

These results help to provide a global understanding of the mechanisms that regulate the complex doping process. Combined with extensive experimental efforts, they should allow for a controlled engineering of defects and dopants for the realization of TCOs with desired electrical character.

## 4. Method

All calculations are performed using density functional theory (DFT) as implemented in the Quantum ESPRESSO (QE) suite of codes [38]. Perdew-Burke-Ernzerhof (PBE) [39] generalized gradient approximation is used for the exchange–correlation functional; single particle wavefunctions (charges) are expanded on a planewave basis set up to a kinetic energy cutoff of 28 Ry (280 Ry). Ionic potential for each chemical species is described by ultrasoft pseudopotentials of the Vandebilt type [40].

Each system is simulated in an orthorombic periodic supercell of size (13.15×11.39×10.63) Å${}^{3}$, which includes 32 unit cells of wurtzite ZnO (i.e., 128 atoms), one Li-X impurity (Li, Li${}_{2}$, LiH, Li-OH, Li-LiO, Li-N, Li-NH${}_{2}$, Li-NO${}_{2}$, Li-CN, Li-F, Li-Cl, Li-I) and—depending on the system—a structural point defect, namely, an oxygen (V${}_{O}$), a zinc (V${}_{Zn}$) or a Zn-O dimer (V${}_{dim}$) vacancy (Figure 1). In order to sample the chemical environment felt by the Li compounds in the ZnO matrix, each system undergoes a cycle of annealing to 300 K for 1.5 ps. The annealing is simulated using the cp.x code of QE that implements the Car–Parrinello [41,42] Lagrangian equations of motion. Then, the systems are cooled down to T = 0 K and the ground state configurations are reached through the total-energy-and-forces optimization approach implemented in the pw.x code. In the latter case, the Brillouin zone integrations are performed using a (4×4×4) Monkhorst–Pack k-point grid, while only Γ-point is used in the Car–Parrinello runs. All structures are relaxed until forces on all atoms are lower than 0.03 eV/Å. The formation enthalpy of single constituents is calculated from the total energy of the optimized reference compounds.

The ground state electronic structures are corrected by including a Hubbard-like potential on both 3d orbitals of zinc (U${}_{Zn}$ = 12.0 eV) and 2p orbitals of oxygen (U${}_{O}$ = 6.5 eV), resulting from a pseudohybrid Hubbard implementation of the DFT+U approach (namely, ACBN0 [43]) as calculated in the AFLOWπ medium frame to high-throughput ab initio calculations [44]. ACBN0 has been demonstrated to profitably correct the energy bandgap as well as the dielectric and vibrational properties of semiconductors and metal oxides [43,45]. In particular, the numerical details and accuracy tests on the effects of the DFT+U approach on the structural and electronic properties of ZnO are reported in previous papers [20,46,47]. The inclusion of Li-based impurities does not change the U values of the pristine host systems. Hubbard corrections for the other chemical species are negligible for all compounds.

For the evaluation of the chemical potential μ(Li,X), we performed a set of total energy calculations for stable phases of Li and X, which includes both solids and/or molecular systems [36].

## 5. Conclusions

We presented an extensive first principles investigation of the effects of Li doping in ZnO. Our results point out the crucial role plaid by structural defects both in the choice of Li absorption site and in the resulting electrical properties of the doped compound. We further analyzed the electronic and optical modifications induced by the simultaneous Li-X co-doping of ZnO. We proved that LiF-doped ZnO is the only codoped system that exhibits a p-type character in the presence of Zn vacancies. Our results explain the amphoteric behavior of Li in ZnO observed in several experimental reports and confirm that the realization of ZnO-based p-type TCOs is still an unsolved scientific challenge.

## Abbreviations

The following abbreviations are used in this manuscript:

TCO	Transparent Conductive Oxide
LXZO	Li-X Doped ZnO
DFT	Density Functional Theory
DOS	Density of States
VBM	Valence Band Maximum
CBM	Conduction Band Minimum
E${}_{g}$	Energy Band Gap
E${}_{F}$	Fermi Level
CNL	Charge Neutrality Level

## References

[1] Stadler A. (2012). Transparent Conducting Oxides: An Up-To-Date Overview. Materials.

[2] Ellmer K. (2012). Past achievements and future challanges in the development of optically transparent electrodes. Nat. Photonics.

[3] Granqvist C.G. (2007). Transparent conductors as solar energy materials: A panoramic review. Sol. Energy Mater. Sol. Cells.

[4] Hautier G., Miglio A., Waroquiers D., Rignanese G.M., Gonze X. (2014). How Does Chemistry Influence Electron Effective Mass in Oxides? A High-Throughput Computational Analysis. Chem. Mater..

[5] King P.D.C., Veal T.D. (2011). Conductivity in transparent oxide semiconductors. J. Phys. Condens. Matter.

[6] Zhang K.H.L., Xi K., Blamire M.G., Egdell R.G. (2016). P-type transparent conducting oxides. J. Phys. Condens. Matter.

[7] Hautier G., Miglio A., Ceder G., Rignanese G.M., Gonze X. (2013). Identification and design principles of low hole effective mass p-type transparent conducting oxides. Nat. Commun..

[8] Sarmadian N., Saniz R., Partoens B., Lamoen D. (2016). Easily doped p-type, low hole effective mass, transparent oxides. Sci. Rep..

[9] Lu J.G., Zhang Y.Z., Ye Z.Z., Zheng Y.J., He H.P., Zhu L.P., Huang J.Y., Wang L., Yuan J., Zhao B.H. (2006). Control of p- and n-type conductivities in Li-doped ZnO thin films. Appl. Phys. Lett..

[10] Lu J., Fujita S., Kawaharamura T., Nishinaka H. (2007). Roles of hydrogen and nitrogen in p-type doping of ZnO. Chem. Phys. Lett..

[11] Zeng Y.J., Ye Z.Z., Xu W.Z., Li D.Y., Lu J.G., Zhu L.P., Zhao B.H. (2006). Dopant source for formation of p-type ZnO: Li acceptor. Appl. Phys. Lett..

[12] Moe Borseth T., Tuomisto F., Christensen J.S., Skorupa W., Monakhov E.V., Svensson B.G., Kuznetsov A.Y. (2004). Deactivation of Li by vacancy clusters in ion-implanted and flash-annealed ZnO. Phys. Rev. B.

[13] Moe Borseth T., Tuomisto F., Christensen J.S., Monakhov E.V., Svensson B.G., Kuznetsov A.Y. (2008). Vacancy clustering and acceptor activation in nitrogen-implanted ZnO. Phys. Rev. B.

[14] Johansen K.M., Zubiaga A., Makkonen I., Tuomisto F., Neuvonen P., Knutsen K.E., Monakhov E.V., Kuznetsov A.Y., Svensson B.G. (2011). Identification of substitutional Li in n-type ZnO and its role as an acceptor. Phys. Rev. B.

[15] Chang J., Lin Z., Zhu C., Chi C., Zhang J., Wu J. (2013). Solution-Processed LiF-Doped ZnO Films for High Performance Low Temperature Field Effect Transistors and Inverted Solar Cells. ACS Appl. Mater. Interfaces.

[16] Ghosh S., Khan G.G., Varma S., Mandal K. (2012). Influence of Li-N and Li-F co-doping on defect-induced intrinsic ferromagnetic and photoluminescence properties of arrays of ZnO nanowires. J. Appl. Phys..

[17] Cao L., Zhu L., Li Y., Yang M., Ye Z. (2012). A facile route to realize p-type ZnO thin films via Li–F codoping: Experiments and theory. Mater. Lett..

[18] Lin B., Fu Z., Jia Y. (2001). Green luminescent center in undoped zinc oxide films deposited on silicon substrates. Appl. Phys. Lett..

[19] Janotti A., Van de Walle C.G. (2007). Native point defects in ZnO. Phys. Rev. B.

[20] Catellani A., Ruini A., Calzolari A. (2015). Optoelectronic properties and color chemistry of native point defects in Al:ZnO transparent conductive oxide. J. Mater. Chem. C.

[21] Wang X.J., Vlasenko L.S., Pearton S.J., Chen W.M., Buyanova I.A. (2009). Oxygen and zinc vacancies in as-grown ZnO single crystals. J. Phys. D Appl. Phy..

[22] Yi J.B., Lim C.C., Xing G.Z., Fan H.M., Van L.H., Huang S.L., Yang K.S., Huang X.L., Qin X.B., Wang B.Y. (2010). Ferromagnetism in Dilute Magnetic Semiconductors through Defect Engineering: Li-Doped ZnO. Phys. Rev. Lett..

[23] Bazzani M., Neroni A., Calzolari A., Catellani A. (2011). Optoelectronic properties of Al:ZnO: Critical dosage for an optimal transparent conductive oxide. Appl. Phys. Lett..

[24] Wang X., Yao B., Shen D., Zhang Z., Li B., Wei Z., Lu Y., Zhao D., Zhang J., Fan X. (2007). Optical properties of p-type ZnO doped by lithium and nitrogen. Solid State Commun..

[25] Johansen K.M., Zubiaga A., Tuomisto F., Monakhov E.V., Kuznetsov A.Y., Svensson B.G. (2011). H passivation of Li on Zn-site in ZnO: Positron annihilation spectroscopy and secondary ion mass spectrometry. Phys. Rev. B.

[26] Wardle M.G., Goss J.P., Briddon P.R. (2005). Theory of Li in ZnO: A limitation for Li-based p-type doping. Phys. Rev. B.

[27] Zhang Y., Lu J., Chen L., Ye Z. (2007). Properties of N-doped ZnO thin films in annealing process. Solid State Commun..

[28] Tarun M.C., Iqbal M.Z., McCluskey M.D. (2011). Nitrogen is a deep acceptor in ZnO. AIP Adv..

[29] Kobayashi K., Tomita Y., Maeda Y., Haneda H. (2008). Shallow Li-acceptor levels in ZnO films codoped with Li and F atoms. Phys. Status Solidi C.

[30] Wang W., Li Z., Liu L., Zhang H., Zheng W., Wang Y., Huang H., Wang Z., Wang C. (2009). Humidity sensor based on LiCl-doped ZnO electrospun nanofibers. Sens. Actuators B Chem..

[31] Chang J., Lin Z., Lin M., Zhu C., Zhang J., Wu J. (2015). Solution processed F doped ZnO (ZnO:F) for thin film transistors and improved stability through co-doping with alkali metals. J. Mater. Chem. C.

[32] Choi Y.J., Kang K.M., Park H.H. (2015). Anion-controlled passivation effect of the atomic layer deposited ZnO films by F substitution to O-related defects on the electronic band structure for transparent contact layer of solar cell applications. Sol. Energy Mater. Sol. Cells.

[33] Choi Y.J., Gong S.C., Kang K.M., Park H.H. (2014). Enhanced hole injection into indium-free organic red light-emitting diodes by fluorine-doping-induced texturing of a zinc oxide surface. J. Mater. Chem. C.

[34] Wang F.H., Yang C.F., Lee Y.H. (2015). Deposition of F-doped ZnO transparent thin films using ZnF2-doped ZnO target under different sputtering substrate temperatures. Nanoscale Res. Lett..

[35] Liu B., Gu M., Liu X., Huang S., Ni C. (2010). First-principles study of flourine-doped zinc oxide. Appl. Phys. Lett..

[B36-materials-10-00332] 36.While PBE XC is a good choice for the characterization of the electronic structure of solids, it is not as accurate as LDA in the calculation of the formation enthalpies. Here, we use PBE results to discuss the relative stability of dopants in ZnO.The exact evaluation of the formation enthalpies for all the Li-X compounds goes beyond the goal of this work, that is instead more focused on the electrical character of the LXZO systems.

[37] Liu L., Mei Z., Tang A., Azarov A., Kuznetsov A., Xue Q.K., Du X. (2016). Oxygen vacancies: The origin of *n*-type conductivity in ZnO. Phys. Rev. B.

[38] Giannozzi P., Baroni S., Bonini N., Calandra M., Car R., Cavazzoni C., Ceresoli D., Chiarotti G.L., Cococcioni M., Dabo I. (2009). Quantum ESPRESSO: A modular and open-source software project for quantum simulations of materials. J. Phys. Condens. Matter.

[39] Perdew J.P., Burke K., Ernzerhof M. (1996). Generalized Gradient Approximation Made Simple. Phys. Rev. Lett..

[40] Vanderbilt D. (1990). Soft self-consistent pseudopotentials in a generalized eigenvalue formalism. Phys. Rev. B.

[41] Car R., Parrinello M. (1985). Unified Approach for Molecular Dynamics and Density-Functional Theory. Phys. Rev. Lett..

[42] Pastore G., Smargiassi E., Buda F. (1991). Theory of ab initio molecular-dynamics calculations. Phys. Rev. A.

[43] Agapito L.A., Curtarolo S., Buongiorno Nardelli M. (2015). Reformulation of DFT+U as a Pseudohybrid Hubbard Density Functional for Accelerated Materials Discovery. Phys. Rev. X.

[44] Supka A.R., Lyons T.E., Liyanage L., D’Amico P., Orabi R.A.R.A., Mahatara S., Gopal P., Toher C., Ceresoli D., Calzolari A. (2017). AFLOW*π*: A minimalist approach to high-throughput ab initio calculations including the generation of tight-binding hamiltonians. arXiv.

[45] Gopal P., Fornari M., Curtarolo S., Agapito L.A., Liyanage L.S.I., Nardelli M.B. (2015). Improved predictions of the physical properties of Zn- and Cd-based wide band-gap semiconductors: A validation of the ACBN0 functional. Phys. Rev. B.

[46] Calzolari A., Ruini A., Catellani A. (2011). Anchor Group versus Conjugation: Toward the Gap-State Engineering of Functionalized ZnO(1010) Surface for Optoelectronic Applications. J. Am. Chem. Soc..

[47] Calzolari A., Nardelli M.B. (2013). Dielectric properties and Raman spectra of ZnO from a first principles finite-differences/finite-fields approach. Sci. Rep..

